# Machine Learning-Based Prediction of Tribological Properties of Epoxy Composite Coating

**DOI:** 10.3390/polym17030282

**Published:** 2025-01-22

**Authors:** Han Yan, Junling Tan, Hui Chen, Tao He, Dezhi Zeng, Lin Zhang

**Affiliations:** School of Mechanical and Electrical Engineering, Chengdu University of Technology, Chengdu 610059, China; thebestyanhan@cdut.edu.cn (H.Y.); 18382273900@163.com (J.T.); 18783477051@163.com (H.C.); m18613280121@163.com (T.H.); zengdezhi0823@163.com (D.Z.)

**Keywords:** machine learning, tribological property, gradient boosting regression, sericite/epoxy composite coating

## Abstract

Machine learning, being convenient and nondestructive, is beneficial for evaluating the tribological properties of coatings. Here, six machine learning algorithms, using a sericite/epoxy composite coating (SEC) as an example, were employed to assess the impact of filler content (10, 15, 20, 25, and 30 wt%) and mesh size on the tribological properties of epoxy composite coatings under different loads. The results showed that the gradient boosting regression model had superior accuracy and stability compared to the other regression models, achieving friction coefficient and wear rate prediction accuracies of 93.7% and 85.7%, respectively. This model outperformed others, including decision trees, extreme gradient boosting, and Gaussian process regression. Feature importance showed that the content of sericite had the most significant influence on the tribological properties. This work provides valuable guidance for the engineering application of this material.

## 1. Introduction

In contemporary sectors such as machinery, geological exploration, petroleum energy, transportation, and other industries, friction and wear phenomena are inevitable during the operation of equipment. These phenomena not only reduce the performance of equipment and shorten its service life but also lead to an increase in the energy consumption of the equipment’s components, which in turn leads to higher maintenance costs [1,2,3,4,5]. Thus, in order to reduce the negative impact of these phenomena, it is crucial to select materials with excellent tribological properties. Coating materials are often preferred as anti-friction and wear-resistant materials because of their high cost-effectiveness, strong environmental adaptability, adjustable performance, and excellent tribological properties [6,7,8,9,10,11]. Traditionally, the tribological properties of coatings have been characterized using experimental methods, such as tensile testing, bending testing, wear testing, etc. While these methods can be effective, they tend to be destructive and time-consuming. Therefore, a convenient, intelligent, and nondestructive method should be developed to assist in predicting the tribological properties of coatings.

Currently, machine learning (ML), which has been extensively applied across sectors including material chemistry, transportation, and mechanical engineering, also holds significant importance within the domain of tribology [12,13,14,15,16]. In the field of material tribology, traditional analysis methods are often insufficient because of the complex nonlinear correlations involved. ML algorithms, with their ability to deal with complex nonlinear relationships, have become important tools in tribological research. Due to its adaptability and efficient data processing ability, ML can predict the tribological properties of materials quickly and nondestructively. At the same time, when addressing challenges in related engineering fields such as petroleum engineering, transportation, and mechanical engineering, ML demonstrates great potential for solving complex nonlinear problems and effectively bridges the gap between theoretical research and practical industrial needs [17,18]. Typically, researchers build predictive models by selecting a variety of ML methods to characterize the typical friction parameters of or conditions faced by the material. Hasan et al. established a variety of ML models to predict the friction and wear of aluminum-based alloys. The study showed that the random forest model performed well in predicting the wear rate, and the K-nearest neighbor model performed the best in predicting the coefficient of friction (COF). Furthermore, the analysis of feature significance indicated that factors such as load, hardness, and sliding velocity were the most critical in forecasting the wear rate of aluminum alloys. In contrast, alloy hardness and the distance slid were identified as the primary determinants in estimating COF [19]. Similarly, Fatih et al. conducted research on the wear characteristics of ZK60 magnesium-based composites reinforced with CeO_2_ contents of 0.25%, 0.5%, and 1 wt% under a range of load conditions and sliding velocities, uncovering the wear mechanisms through experimental studies. In addition, five machine learning algorithms, namely random forest, XGBoost, decision tree, support vector regression, and multi-layer perceptron, were used to predict the wear behavior of the material. By comparing the predicted results, it was concluded that the tree-based algorithm model showed the best prediction performance, and the load was the most important factor affecting many of the characteristic parameters of volume loss measurement [20]. Wang et al. analyzed the friction and wear properties of surfaces with MoS_2_ coatings under varying load conditions and frequencies and explored the wear mechanism. After successfully predicting the tribological properties using the gradient boosting regression tree algorithm, it was also determined that the load was the most influential parameter affecting the COF and wear rate of the coating [21]. Their research results not only validated the great potential of combining tribological experiments with machine learning techniques for predicting the tribological properties of composite materials but also provided a more intelligent, efficient, and high-precision approach for the study of the friction and wear behavior of composite materials. Despite the positive results of these studies, selecting the most appropriate ML algorithm for predicting the tribological properties of materials in experiments remains a challenge. Not all ML algorithms are suitable for the study of the tribological properties of materials. Therefore, when selecting a suitable ML algorithm, it is necessary to consider not only the quality and quantity of the dataset used but also the scenarios applicable to the ML algorithm. Generally, the sample size and the number of features in the dataset have a great impact on the overall prediction performance of the ML model [22].

In order to determine the appropriate model, various machine learning algorithms must be tested, the best algorithm must be selected through model training, and then that must be used to predict the tribological properties of the material. The training accuracy of the model is affected not only by the adjustment of the internal parameters but also by the selection of feature parameters in the dataset, which plays a crucial role in improving training accuracy.

Most studies have focused on parameters such as temperature, frequency, and sliding velocity under friction conditions or have obtained parameters through methods such as molecular dynamics and then used ML to study materials’ properties [23,24,25]. However, research taking the inherent properties of fillers as characteristic parameters to study their impact on the tribological performance of coatings remains relatively limited. Moreover, the effective selection of algorithms and the accuracy of predicting the tribological performance of coatings have not been extensively studied. Sericite, a widely used two-dimensional filler material, is popular in industrial applications for its excellent mechanical and chemical properties [26]. Based on this, sericite was incorporated into an epoxy matrix to form a composite coating, and its tribological properties are evaluated using ML techniques in this study. Specifically, six ML methods, namely random forest (RF), gradient boosting regression (GBR), Gaussian process regression (GPR), an artificial neural network (ANN), support vector regression (SVR), and extreme gradient boosting (XGB), were selected to predict the tribological performance of sericite/epoxy composite coatings (SECs). The input parameters for these models included load, filler content, and mesh size, and the fitting performance of each model was compared. Ultimately, the ML model with the highest fitting accuracy was selected, and the influence of different characteristic parameters on tribological performance was analyzed through the feature importance index of the model. The results of this study provide valuable insights into the friction and wear behavior of SEC and provide guidance for the engineering application of this material.

## 2. Experimental Section

### 2.1. Preparation of Materials

Epoxy resin (H268A) and the corresponding curing agent (H268B) were purchased from Shanghai Hanzhong Chemical Co., Ltd. (Shanghai, China). A high-speed mixer was purchased from Shanghai Bei Micro Motor Co., Ltd. (Shanghai, China). Sericite powder was purchased from Henan Zhengzhou Huifeng New Material Co., Ltd. (Zhengzhou, China). Two solvents, acetone and ethanol, were supplied by Chengdu Colon Chemical Co., Ltd. (Chengdu, China). The deionized water used in all experiments was prepared by the deionized water mechanism.

### 2.2. Preparation of Sericite/Epoxy Composite Coating

**Figure 1:**
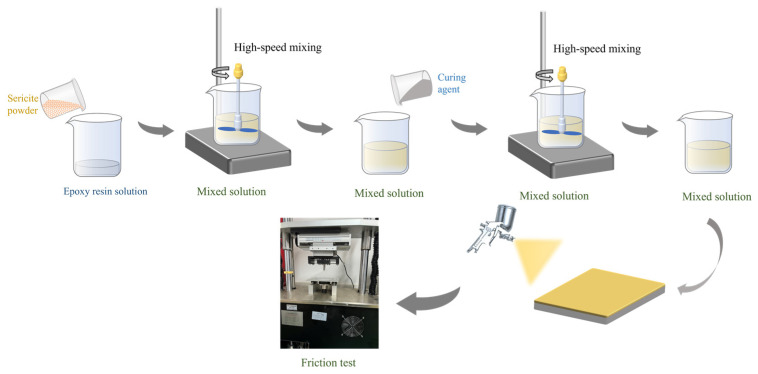
Coating preparation flow chart.

Steel templates (Q235) with a size of 1.5 cm × 1.5 cm × 0.1 cm were polished using sandpaper with a mesh size of 400, 800, and 1200 in turn. Each plate was then ultrasonic-cleaned in a solution of acetone and ethanol to remove any remaining stains on the surface. The preparation process of the coating is shown in Figure 1. First, the epoxy resin and the curing agent were prepared according to a mass fraction ratio of 2:3 to form an epoxy resin solution. After that, sericite powder with different mesh sizes (325, 500, and 800 mesh) and weight contents (10, 15, 20, 25, and 30 wt%) was added to the epoxy resin solution and stirred at 3500 rpm for 5 min. Then, the curing agent was added to the mixture and stirred at 2500 rpm for 5 min. Finally, the mixture was left to stand for a period of time in a vacuum environment with negative pressure to eliminate bubbles. The resulting slurry was then uniformly sprayed onto the surface of the steel plate, where it cured at room temperature for 48 h to form the final sericite/epoxy composite coating (SEC). The coating had a thickness of about 35 μm.

### 2.3. Characterization of Tribological Properties

A ball-and-disc friction pair was used to investigate the tribological properties of the coating materials. The friction test was carried out on a high-speed reciprocating friction test machine produced by Friction Equipment Co., Ltd. (Kyoto, Japan), East China Jiaotong University, Jiangxi, China, as shown in Figure 1. Prior to the experiment, anhydrous ethanol was used to clean both the sample and the indenter, removing any grease and impurities from the surface. Meanwhile, all friction tests were conducted at a constant temperature (25 °C) and a fixed test duration (60 min). The friction counterpart for the coating was a GCr15 bearing steel ball with a diameter of 6 mm. The reciprocating frequency was set to 2 Hz, and the tribological performance tests were conducted on epoxy coatings containing different contents of sericite (10, 15, 20, 25, and 30 wt%) under varying loads (5, 10, and 15 N). Scanning electron microscopy (SEM, Thermo Fisher Quattro S, Waltham, MA, USA) was used to characterize the morphology of the coating sample, and a white light interferometer (VK-X 5000, Keyence, Osaka, Japan) was used to observe the three-dimensional morphology and wear marks of the worn surface. The distribution of elements within the coating was analyzed using an energy-dispersive spectrometer (EDS). To ensure the accuracy and stability of the results, each group of friction experiments was repeated three times under identical conditions.

### 2.4. Machine Learning Algorithm

By applying six ML models (RF, GBR, GPR, ANN, SVR, and XGB), the nonlinear relationship between the feature parameters and the target parameters was established. The best model for predicting tribological performance was selected through a comparative analysis of their performances. The ML models were implemented in a Python 3 environment using the open-source scikit-learn package. A brief description of the six ML methods used in this study is provided below.

**Figure 2:**
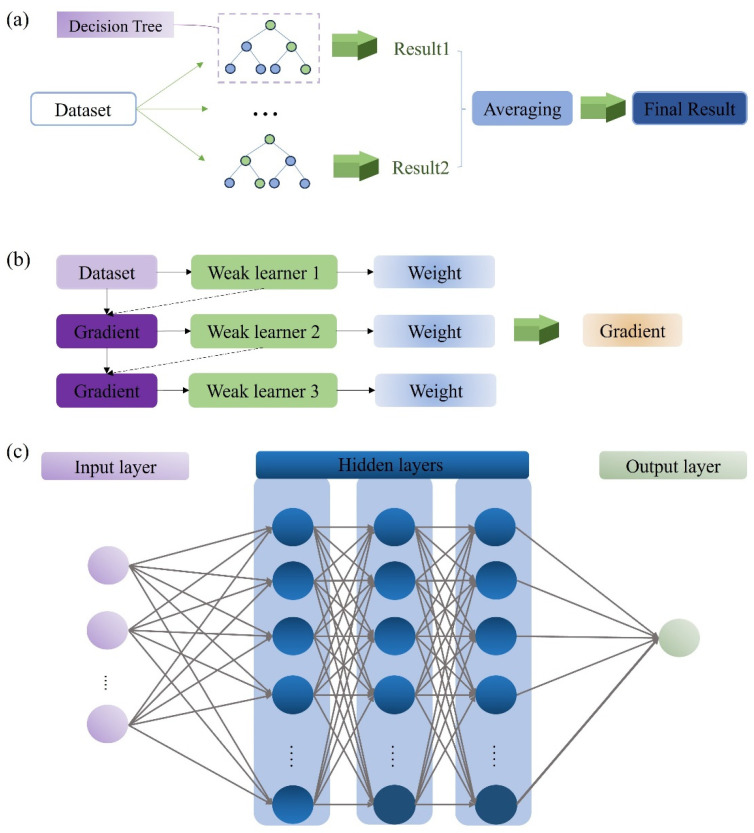
Structure graphs of (**a**) random forest, (**b**) gradient boosting regression, and (**c**) an ANN.

RF is an ensemble learning algorithm commonly used for both classification and regression problems. RF consists of multiple decision trees, each constructed using randomly drawn samples from the training data and a randomly selected subset of features. The final result is determined by voting or averaging across these trees. This approach effectively reduces the correlation between individual trees and enhances the model’s overall prediction accuracy and generalization ability. A schematic of the RF model is shown in Figure 2a [27,28,29].

GBR is a boosting-based algorithm that generates a series of weak learners sequentially. Each weak learner fits the residuals of the previous model’s predictions, and through various combination strategies, these weak learners are iteratively refined to create a stronger model [30]. The structure of the model is shown in Figure 2b. The GBR model was found to perform well in predicting the COF and wear rate of composites in previous research on the tribological properties of composites [31,32].

GPR is a non-parametric model known for its computational simplicity and ease of use. It enables powerful statistical computations within a Bayesian framework, which not only allows more efficient uncertainty estimation in predictions but also transforms the model selection process into solving a nonlinear optimization problem. Gaussian process regression mainly makes use of kernel functions (e.g., the Gaussian covariance function (RBF), exponential class of covariance functions, polynomial covariance functions, sigmoid kernel, etc.) to construct covariance matrices between all pairs of data and learn a nonlinear mapping from input features to true value outputs [33].

ANNs are computational models composed of multiple neurons that pass information to one another. The structure of the ANN model is shown in Figure 2c. It consists of three different parts: an input layer, one or more hidden layer, and an output layer. Each component consists of one or more neurons that process incoming information through an activation function before transmitting it to the next neuron. The accuracy of the model depends on several key parameters, including the number of hidden layers, the activation function used, and the number of neurons [34].

SVR is an application of support vector machines for dealing with regression problems. It is mainly used to predict output values, with its core principle being the minimization of the error between predicted values and actual observed values within a specified tolerance range. This is achieved by finding a kernel function while ensuring the function remains as smooth as possible. SVR can provide a better prediction performance than traditional linear regression when dealing with nonlinear relation problems [35].

XGB is an efficient algorithm based on the gradient lifting decision tree. It controls the complexity of the model by adding regularization terms to the loss function, effectively preventing overfitting and thus improving the generalization ability of the model. In regression problems, XGB can efficiently handle large datasets [36].

### 2.5. Performance Evaluation of ML Models

Evaluation metrics are used to quantify deviations between the predictions of a machine learning model and actual observations, helping to evaluate the model’s fit to the dataset. For regression models, key evaluation indicators include the R-squared coefficient (*R*^2^), mean squared error (MSE), root mean squared error (RMSE), and mean absolute error (MAE) [37].

MSE represents the average squared difference between the model’s predicted value and the true value. In regression problems, larger errors are given more weight due to the squaring of the differences. The formula is as follows:

(1) $$MSE=\frac{1}{N}\sum_{i=1}^{N}{\left({\hat{y}}_{i}-y_{i}\right)}^{2}$$

RMSE is obtained by taking the square root of the MSE, which provides a measure of error in the same units as the predicted and true values. The formula is as follows:

(2) $$RMSE=\sqrt{\frac{1}{N}\sum_{i=1}^{N}{\left({\hat{y}}_{i}-y_{i}\right)}^{2}}$$

MAE refers to the average absolute difference between the model’s predicted value and the true value. Since the MAE does not square the error, it has less of an effect on outliers. The formula is as follows:

(3) $$MAE=\frac{1}{N}\sum_{i=1}^{N}\left|{\hat{y}}_{i}-y_{i}\right|$$

*R*^2^ is calculated as follows:

(4) $$R^{2}=1-\frac{\sum_{i=1}^{N}{\left({\hat{y}}_{i}-y_{i}\right)}^{2}}{\sum_{i=1}^{N}{\left(\bar{y}-y_{i}\right)}^{2}}$$

where $y_{i}$ is the true value, ${\hat{y}}_{i}$ is the predicted value, $\bar{y}$ is the average of the true values of all samples, and N represents the number of samples. The top half of the *R*^2^ score is the sum of the squared differences between the predicted values and the true values, and the bottom half is the sum of the square differences between the true values and the mean of the true values [38]. It takes values in the interval [0, 1]. *R*^2^ values closer to 0 indicate that the correlation is weaker and the model is insufficient to explain the variation in the data. The closer the *R*^2^ value is to 1, the stronger the correlation is and the more accurately the model can respond to changes in the data [39].

### 2.6. Data Processing

Initially, 45 sets of friction tests were performed to collect information on the COF and wear rate of the coating under varying loads, different material sizes, and different material mixing ratios. The experimental data obtained from the friction and wear tests were compiled as a dataset, with 80% used for model training and 20% used for model testing. The load, material mesh, and material content in the dataset were taken as characteristic parameters, while the COF and wear rate were taken as target parameters. At the same time, the feature parameters in the dataset were subjected to data normalization. In addition, to prevent overfitting of the model, all the hyperparameters required by the model during training were optimized by grid search and cross-validation methods to optimize the model’s performance.

## 3. Results and Discussion

### 3.1. Influence of Content on the Tribological Properties of SEC

**Figure 3:**
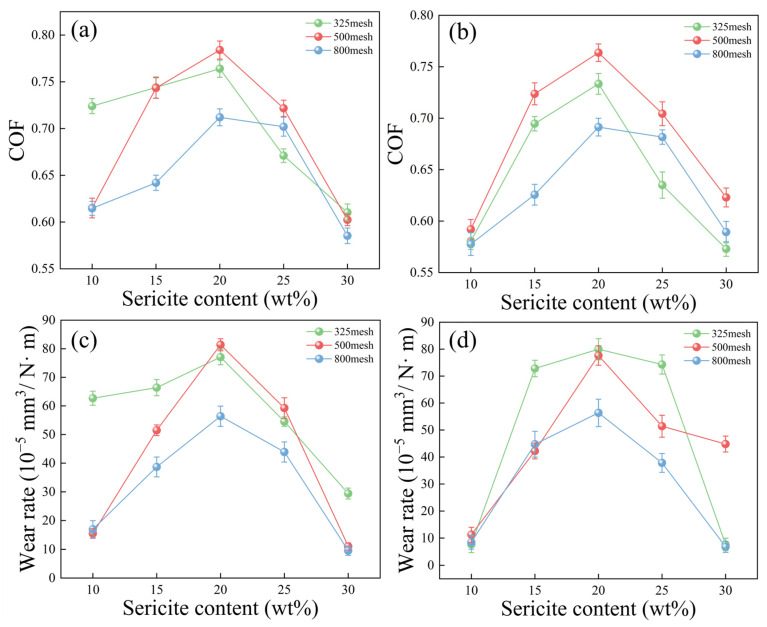
Curve graphs of the COF and wear rate at different loads (10 and 15 N). (**a**,**c**) Curves of changes in the COF and wear rate with sericite content under a load of 10N. (**b**,**d**) Curves of changes in the COF and wear rate with sericite content under a load of 15 N.

**Figure 4:**
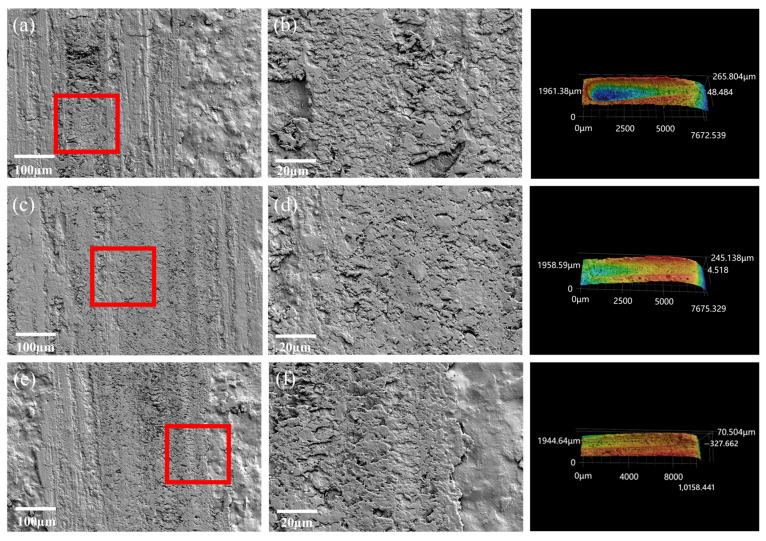
SEM worn surfaces of the coating with a sericite content of 20 wt% under different mesh sizes (325, 500, and 800 mesh) and different loads (5, 10, and 15 N). (**b**,**d**,**f**) SEM images of the enlarged coating wear marks in the red boxes in (**a**,**c**,**e**).

**Figure 5:**
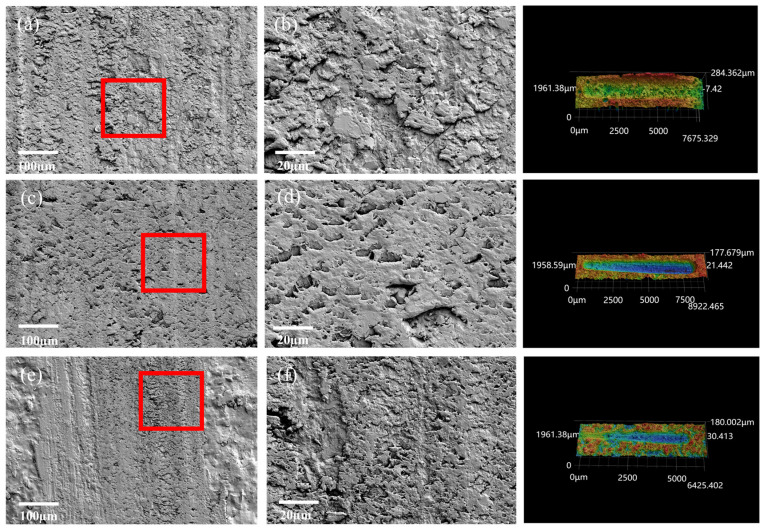
SEM worn surfaces of the coating with a sericite content of 30 wt% under different mesh sizes (325, 500, and 800 mesh) and different loads (5, 10, and 15 N). (**b**,**d**,**f**) SEM images of the enlarged coating wear marks in the red boxes in (**a**,**c**,**e**).

Figure S1 shows the EDS images of the wear surface of the coating material. The analysis results indicated a significant distribution of Si and Al elements from the sericite powder, confirming its good dispersion within the coating [40]. Figure S2 and Figure 3 show the changes in the COF and wear rate under three loads. Figure 4 and Figure 5 show the wear marks on the coating materials observed by SEM at different magnifications. Figure 3a,c indicate that under a load force of 10 N, the COF and wear rate of the SEC changed with different mesh numbers and mass fractions, while Figure 3b,d show the results under a load force of 15 N. It can be observed from the figure that under the same load, the COF and wear rate of the coating material reached a peak when the sericite content was 20 wt%. The SEM observations of the wear marks on the coating material in Figure 4 and Figure 5 show that for the sericite coating with a 20% mass fraction, furrows and accumulation caused by particle shedding appeared on the wear surfaces (Figure 4a,c,e), with varying intensity as the load increased. These phenomena were related to the response behavior of the coating material during friction. Due to the sliding contact between the ball on the friction pair and the contact point of the coating material during the friction process, a shear effect was generated, resulting in coating fragments being removed from one side of the contact point and adhering to the convex body on the other side. As the sliding continued, the shed particles were either removed from their adhesive surface and transferred to the surface of the coating or fell off around it, exhibiting a typical adhesive wear characteristic. The damage mechanism of the coating material led to an increase in its wear rate and COF. According to the SEM images in Figure 4b,d,f, the wear debris on the wear surface was evenly distributed and had a smooth adhesion surface. In contrast, in Figure 5b,d,f, the wear debris on the substrate surface of the SEC coating with a sericite content of 30 wt% showed more uneven and loose accumulation, resulting in a decrease in the COF and wear rate.

### 3.2. Predicting Tribological Performance of Coatings with ML Models

**Figure 6:**
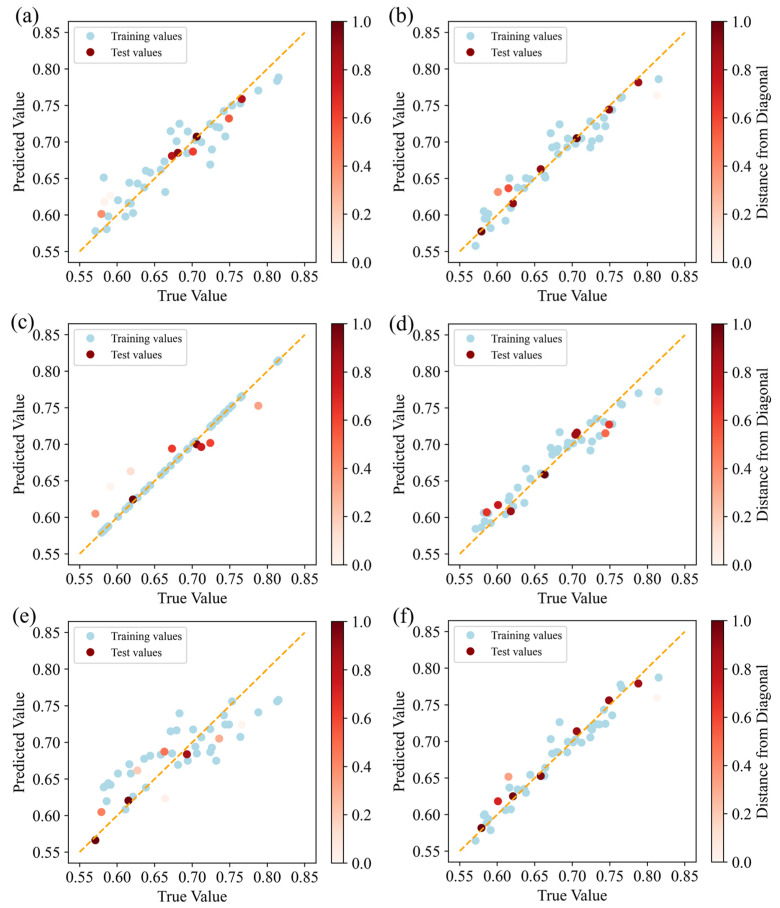
Predictive performance of the friction coefficient using the following models: (**a**) ANN, (**b**) GBR, (**c**) GPR, (**d**) RF, (**e**) SVR, and (**f**) XGB.

Six ML models (ANN, GBR, GPR, RF, SVR, and XGB) were employed to predict and evaluate the impact of fillers in the epoxy composite coating on its tribological performance. Figure 6 andFigure 7 present the comparison between the predicted and actual values for the COF and wear rate datasets, respectively, as predicted by each ML model. In the figures, the true value on the *X*-axis represents the COF or wear rate in the dataset collected through actual experiments, and the predicted value on the *Y*-axis represents the COF or wear rate predicted by the model when fitting the samples in the dataset. The red points represent samples in the test dataset, while the blue points represent samples in the training dataset. The orange dashed line indicates the ideal fitting effect. If all prediction points fall precisely on this line, it would mean that the model’s predictions perfectly match the true observed values. The shade of the red points reflects their deviation from the orange dashed line: the closer a red point is to the orange line, the darker its color, indicating a closer match between the model’s predicted value and the true value. Lighter shades indicate greater deviation, signifying a more pronounced difference between the predicted and true values.

**Figure 7:**
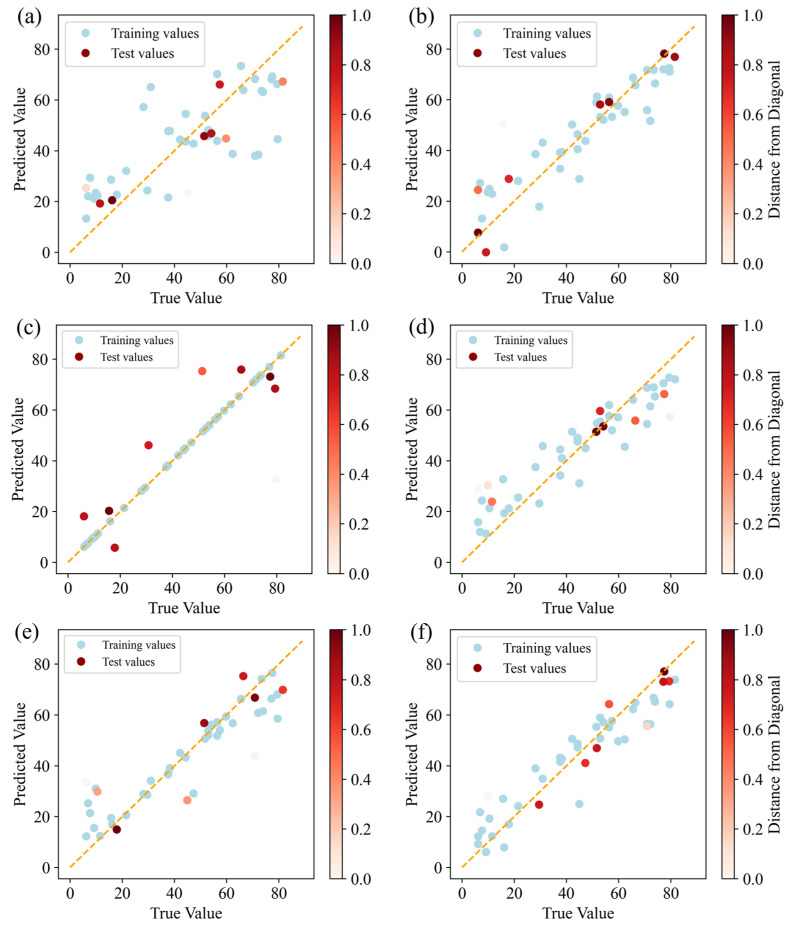
Predictive performance of the wear rate using the following models: (**a**) ANN, (**b**) GBR, (**c**) GPR, (**d**) RF, (**e**) SVR, and (**f**) XGB.

**Figure 8:**
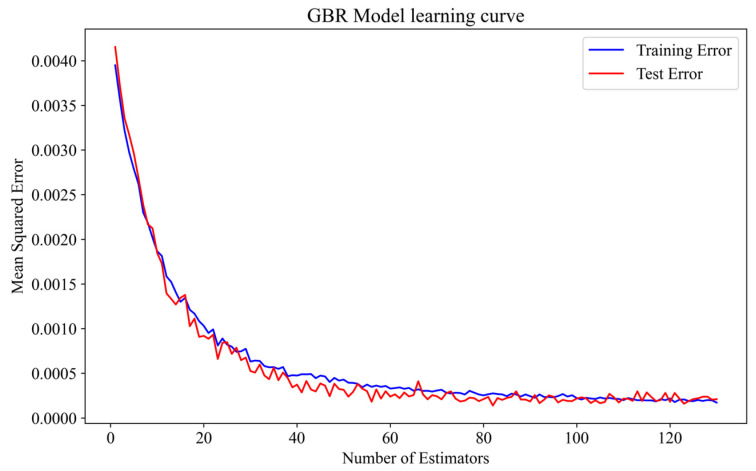
Learning curve of the gradient boosting regression model.

It can be seen from Figure 6 that although the blue points in the GPR model are all on the orange line, this was because, in GPR, the self-covariance of the kernel matrix was zero, and the output value of the training data points was precisely known without uncertainty. Therefore, the model did not produce prediction errors when training on the output points. However, when predicting the true value, uncertainty was introduced, causing the red dots in the result to be biased. The low number of red dots clustered near the orange dotted line in the GPR model indicates that its ability to predict the COF was weaker than that of the other models. As observed from the prediction results in Figure 6a,b,d, there were more red dots clustered along the orange dotted line in the GBR and XGB models than in the other models. This indicates that the GBR and XGB models’ predicted values deviated less from the actual values, and the models could learn the relationship between the input and output variables more accurately. Figure 7 displays the performance of the six ML models in predicting the wear rate. It can clearly be seen that the GBR model has the largest number of red dots near the orange dotted line, indicating that the deviation between the predicted values and actual values in the GBR model was relatively small. The XGB model had fewer red dots near the orange dotted line than the GBR model. Therefore, the GBR model was superior to the other learning models in predicting the wear rate. In addition, to reduce the influence of overfitting or underfitting on the model’s prediction accuracy, Figure 8 shows the trend of training and test errors in our GBR model as the number of base learners increased. In the figure, the vertical axis presents the MSE, and the horizontal axis presents the number of estimators. It was observed that both training and test errors decreased gradually with the increase in the number of estimators. After the number of estimators reached approximately 20, the error decreased significantly and began to converge, eventually becoming stable within a certain range, indicating that the model did not exhibit obvious overfitting.

**Figure 9:**
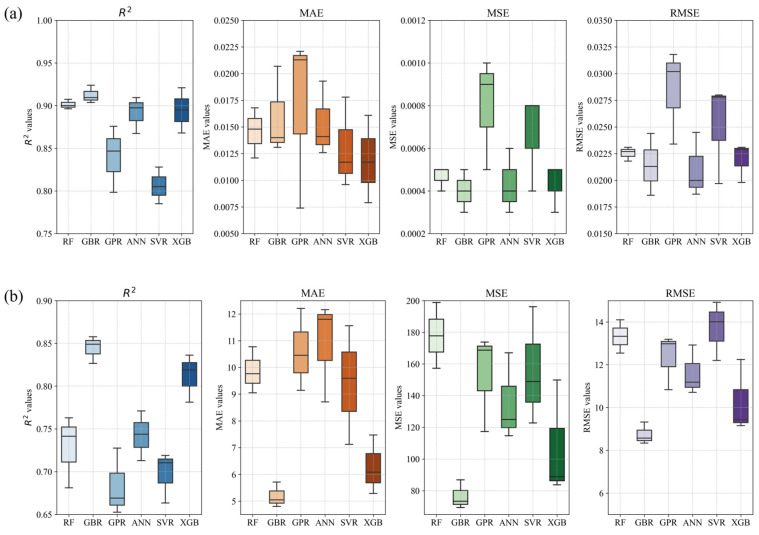
Performance comparison of different models based on evaluation indicators: (**a**) COF and (**b**) wear rate.

The evaluation metrics for the COF and wear rate for the six models are presented in Table 1 and Table 2, respectively. Table 1 shows that the *R*^2^ values of the RF, GBR, GPR, ANN, SVR, and XGB models were 0.908, 0.937, 0.876, 0.91, 0.828, and 0.921 respectively. The GBR model had the highest *R*^2^ value, with corresponding MAE and MSE values of 0.0051 and 0.0003. In terms of wear rate prediction accuracy, as seen in Table 2, the *R*^2^ values of the RF, GBR, GPR, ANN, SVR, and XGB models were 0.741, 0.857, 0.728, 0.771, 0.719, and 0.836, respectively. The GBR model achieved the highest *R*^2^ and had the lowest values of MSE, RMSE, and MAE compared to the other models. Figure 9 compares the evaluation indexes of the six ML models applied in this study in terms of predictive performance to assess the stability and accuracy of their predictive results on the training set. The results show that compared to the other models, the GBR model had a stronger and more stable predictive ability. This was precisely because the GBR model gradually optimized the residuals generated each time in the iterative process, so that each newly trained tree focused on correcting the error of the previous tree. Additionally, GBR allows parameters such as the learning rate, number of trees, and depth of trees to be adjusted, which allows better control over the model fit and complexity. Therefore, the GBR model was more suitable for predicting the friction and wear characteristics of the composite coating material.

**Figure 10:**
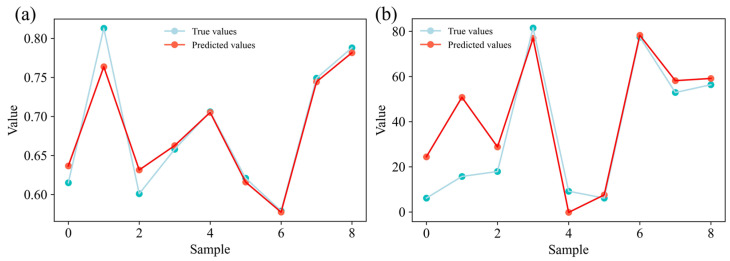
Predicted vs. true values of (**a**) COF and (**b**) wear rate.

Figure 10 shows the application of the GBR model, which demonstrated superior performance in predicting the COF and wear rate, to forecast values from the experimental data. The results show a strong agreement between the predicted and actual values for both the COF (Figure 10a) and wear rate (Figure 10b). Although the GBR model’s prediction results were slightly different from the actual values at some data points, the overall trend shows that the model successfully captured changes in the tribological properties of SEC materials. The consistency between the predicted and actual values highlights the model’s performance in terms of predictive accuracy. The model’s ability to predict the SEC material’s properties can significantly reduce both the time and cost required for actual experiments during the research process. These benefits position the GBR model as a powerful tool for predictive analysis in tribological studies, providing valuable insights into material performance without the need for extensive experimental trials, and this model could also provide important guidance for the application of this material in the engineering field.

**Figure 11:**
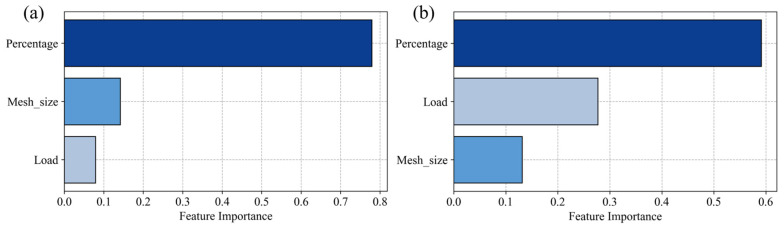
Feature importance for prediction of (**a**) COF and (**b**) wear rate.

In order to further explore the importance of the influence of feature parameters on the prediction ability of the model, Figure 11 shows the contribution of each feature in the GBR model to the prediction of COF and wear rate. Feature importance quantifies the contribution of each input variable (feature parameter) to the model’s prediction results. The higher the importance is, the greater the impact of the feature is on the model’s prediction accuracy. For COF prediction, as shown in Figure 11a, the percentage of sericite filler was the dominant factor influencing the model’s predictive ability. This suggests that changes in sericite content strongly affect the friction behavior of the composite material. In comparison, the mesh size of the filler was slightly more influential than the load, while both parameters had relatively lower importance than the filler percentage. These results indicate that while the load and mesh size did influence the COF, their impact was less pronounced than that of the filler content. In the case of wear rate prediction, as shown in Figure 11b, the feature importance analysis revealed a somewhat different hierarchy. Although the percentage of sericite still held the highest importance, the load exhibited a significantly greater contribution compared to its importance in COF prediction. This highlights that while filler content remained the most critical factor, the load applied during testing had a much stronger impact on the wear rate than on the COF. The mesh size in this case was the least important factor, but it still played a notable role. The results of the two graphs show that there was a strong correlation between the filler percentage and the predicted result, while the correlation between the load and the mesh size and the predicted result was relatively weak.

The feature importance analysis revealed that sericite content was the main factor affecting the predicted COF and wear rate of SEC materials in the GBR model. The results showed that the concentration and dispersion of sericite filler in the matrix largely determined the friction and wear behavior of the material. In addition, the load and mesh number were also identified as key factors affecting the tribological properties of SEC materials, although their effect was relatively smaller than that of the filler content. Therefore, the influence of sericite content on tribological properties should be primarily considered in practical experiments.

## 4. Conclusions

The wear mechanism of SEC materials was analyzed experimentally, and the effects of load and sericite mesh size and content on the tribological properties of the SEC materials were studied using ML models. Based on the data collected in the experiment, six ML models were selected to compare the prediction results of COF and wear. The main conclusions are as follows.

(1) As the sericite content increased, the COF and wear rate of the coating initially increased and then decreased, and the wear mechanism was adhesive wear. The load, sericite content, and mesh size were selected as the characteristic parameters, and six ML algorithms were used to predict the tribological properties of the SEC.
(2) By comparing the evaluation metrics between the six ML models, it could be seen that the GBR model performed very well in terms of prediction accuracy for COF, with a prediction accuracy of 93.7%. At the same time, the model also achieved a satisfactory prediction accuracy for the wear rate, with an accuracy of 85.7%. This result highlighted the potential of the GBR model in the prediction of the tribological properties.
(3) The results of the characteristic importance analysis for the GBR model showed that the percentage of sericite was an important parameter for assessing the tribological properties of the SEC materials. In addition, the mesh number and the load also had some relatively small effects on their COF and wear rates, respectively.
(4) The ML algorithm model can effectively explain the relationship between the filler content and the tribological properties of the coating and provide an important guide for the application of the material in the engineering field.

## Figures and Tables

**Table 1:** Predictive evaluation indexes of each model for the COF.

ML Model	MAE	MSE	RMSE	*R* ^2^
Random forest	0.0121	0.0004	0.0218	0.908
Gradient boosting regression	0.0051	0.0003	0.0209	0.937
Gaussian process regression	0.0213	0.0005	0.0234	0.876
Artificial neural network	0.0141	0.0004	0.0200	0.910
Support vector regression	0.0117	0.0008	0.0281	0.828
Extreme gradient boosting	0.0079	0.0005	0.0229	0.921

**Table 2:** Predictive evaluation indexes of each model for the wear rate.

ML Model	MAE	MSE	RMSE	*R* ^2^
Random forest	10.77	198.85	14.11	0.741
Gradient boosting regression	5.08	69.44	9.32	0.857
Gaussian process regression	9.14	117.37	10.83	0.728
Artificial neural network	12.17	114.66	10.71	0.771
Support vector regression	9.59	148.87	12.20	0.719
Extreme gradient boosting	6.08	83.71	9.15	0.836

## Data Availability

The original contributions presented in this study are included in the article/Supplementary Materials. Further inquiries can be directed to the corresponding author.

## Supplementary Materials

The following supporting information can be downloaded at: https://www.mdpi.com/article/10.3390/polym17030282/s1, Figure S1: The EDS spectrum and elemental distribution map of coatings; Figure S2: Curve graph of the COF and wear rate at 5 N load. (a,b) Curves of changes in the COF and wear rate with sericite content under a load of 5 N.
