# Supplementary material for: Machine Learning-Based Prediction of Tribological Properties of Epoxy Composite Coating
Source: Polymers (Basel). 2025 Jan 22;17(3):282. doi: 10.3390/polym17030282 (PMC11820021; doi:10.3390/polym17030282)
Supplement: Supplementary file 1 [file polymers-17-00282-s001.zip › polymers-3395413-supplementary.pdf]

Machine Learning-Based Prediction of Tribological Properties of Epoxy Composite Coating

Han Yan[1], Junling Tan[1] ,Hui Chen[1] ,Tao He[1], Dezhi Zeng[1], Lin Zhang[1*]

[1] *School of Mechanical and Electrical Engineering, Chengdu University of Technology, Chengdu 610059, China*

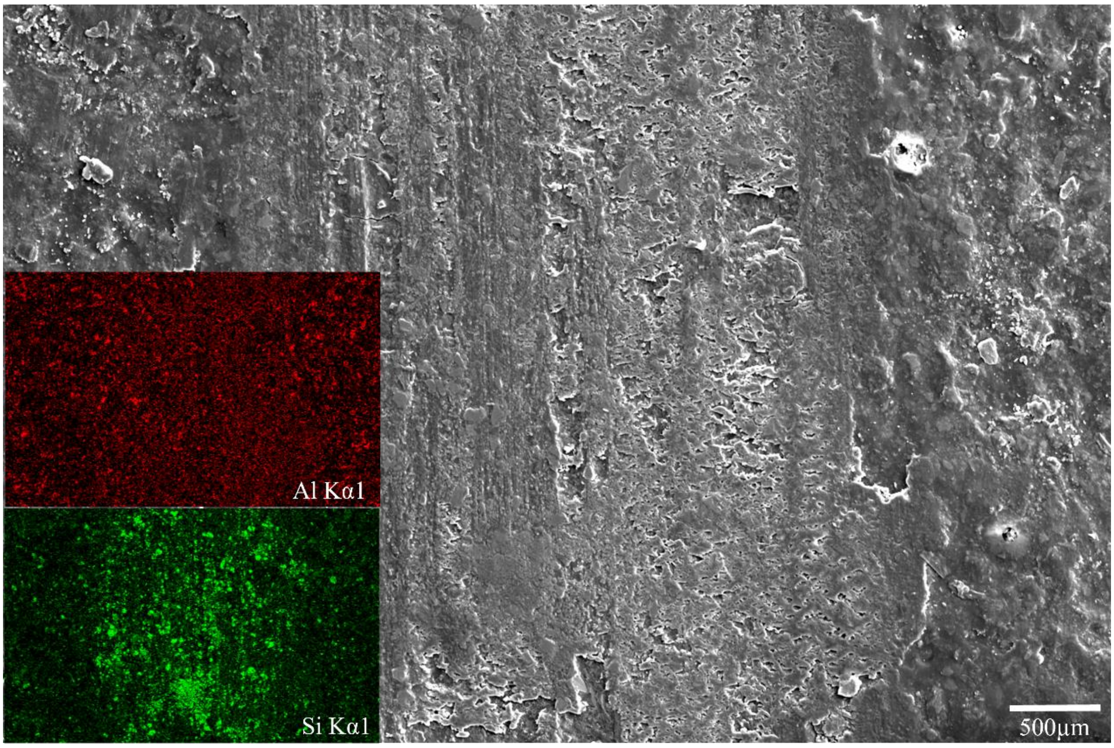

Figure S1 The EDS spectrum and elemental distribution map of coatings.

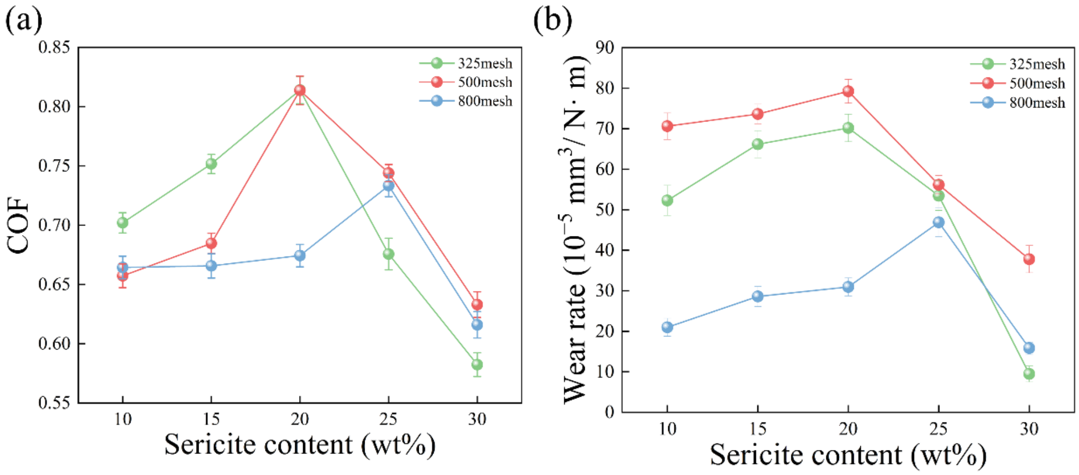

Figure S2 The curve graph of COF and wear rate at 5 N load. Figures (a) and (b) are curves of changes in COF and wear rate with sericite content under a load of 5N.
